# Editorial: Nutritional counseling for lifestyle modification

**DOI:** 10.3389/fnut.2024.1472316

**Published:** 2024-08-16

**Authors:** Cinzia Ferraris, Ramona De Amicis

**Affiliations:** ^1^Laboratory of Food Education and Sport Nutrition, Department of Public Health, Experimental and Forensic Medicine, University of Pavia, Pavia, Italy; ^2^Human Nutrition and Eating Disorder Research Center, Department of Public Health, Experimental and Forensic Medicine, University of Pavia, Pavia, Italy; ^3^International Center for the Assessment of Nutritional Status and the Development of Dietary Intervention Strategies (ICANS-DIS), Department of Food Environmental and Nutritional Sciences (DeFENS), University of Milan, Milan, Italy; ^4^IRCCS Istituto Auxologico Italiano, Obesity Unit and Laboratory of Nutrition and Obesity Research, Department of Endocrine and Metabolic Diseases, Milan, Italy

**Keywords:** nutritional counseling, dietary counseling, lifestyle, dietary habits, nutrition education

Nutritional counseling (NC), as a cornerstone in managing chronic diseases, is increasingly pivotal in promoting healthy lifestyles and mitigating conditions driven by poor dietary habits. It consists of a supportive process, characterized by a collaborative interaction through which clients (i.e. patients) are active participants, together with counselors (i.e. healthcare professionals), in the interpretation and management of their nutritional problems, needs and goals (1–3).

Nutritional counselors serve as crucial mediators in the quest for better lifestyle modification. They provide tailored advice that aligns with individuals' unique needs, facilitating changes that are sustainable and effective. This professional guidance is not a one-way street; it involves active participation from patients, making them integral players in their journey toward better health. The counselors employ techniques that address stress management, enhance motivation, and promote empowerment through education and communication. This comprehensive approach ensures that the dietary changes recommended are not only suitable but also sustainable.

Several systematic reviews in the series highlight the wide range of effects of NC. One such review, by Neri, Mariotti et al., examines dropout rates in cognitive behavioral therapy (CBT), the oldest and most studied behavior change theory used in NC for adults with obesity. It provides insights into the barriers and facilitators to sustained engagement in weight management programs: dropout rates varied (5%−62%) according to common reasons (i.e. long-term illness, acute illness and pregnancy), logistical reasons, poor work conditions or job difficulties, low level of organization, dissatisfaction with initial results, lack of motivation and lack of adherence. However, CBT showed promise with lower dropout rates than non-behavioral interventions.

Another review by Neri, Guglielmetti et al. focuses on the effectiveness of NC in childhood and adolescence, a critical period for the early establishment of healthy lifelong habits, where consistent nutritional intervention to combat obesity and related health problems can make a difference for the rest of the lifetime. This review has shown that various NC approaches can be successful in improving health outcomes in children and adolescents. These approaches have demonstrated positive impacts on a range of factors, including weight management (anthropometric and body composition parameters), dietary habits, nutritional knowledge, physical activity levels. However, implementing NC in this age group requires adapting counseling strategies to suit the evolving cognitive abilities of children at different stages of development. This can be a challenge, but effective strategies do exist. In the same age group, Urrea et al. investigated the relationship between thyroid hormones and weight management in children and adolescents with overweight or obesity. They conducted a scoping review, showing that the changes in body composition parameters in response to the multidisciplinary interventions based on nutritional education and/or counseling correlated positively with fT3, TT3, or TSH.

Original research articles delve into specific interventions and their clinical outcomes. A study by Mambrini et al. introduces the NUTRIKOB questionnaire, designed to assess the nutritional knowledge of hospitalized patients with obesity. This tool exemplifies how targeted educational resources can empower patients to make informed dietary choices, potentially improving their health outcomes. Increasing nutrition knowledge is one of the main objectives of nutrition counseling: it has been shown to be a relevant part of the food choice decision process, along with other factors such as age, gender and socio-economic status. Indeed, nutritional knowledge can influence food choices both indirectly, for example by helping to understand and remember food labels, and directly by influencing consumer behavior.

In another study, Maneesing et al. explore the impact of portioned meal boxes on blood glucose control in patients with type 2 diabetes, conducting a randomized parallel intervention trial over 12 weeks. A general NC with the addition of portioned meal boxes resulted as a valuable tool in dietary management for patients, providing a practical approach to portion control and nutritional balance. This method may enhance adherence to dietary recommendations and improve clinical outcomes, highlighting the potential of structured NC in chronic disease management. Similarly, Oda et al. present results from a retrospective study of the effects of dietary advice on various clinical parameters in patients with obesity. Authors highlighted the broad health benefits of structured dietary counseling in the internal medicine service, even if weight reduction was not significant. This suggests that to improve metabolic health outcomes, it is important to integrate structured dietary advice into the management of moderate obesity.

The importance of tailored NC is further emphasized in research focusing on specific populations. Feng et al. investigate the role of WeChat group management in improving stress adaptation and insulin resistance among women with gestational diabetes mellitus during the COVID-19 pandemic. Women in the WeChat-managed group showed significant reductions in reduced stress hormones, improvements in oxidative stress markers and better insulin resistance. Therefore, this study highlights the potential of digital platforms in extending the reach and effectiveness of NC, particularly during times of limited physical interaction.

NC is not limited to those with chronic conditions. Fiorini et al. review the application of NC among athletes, emphasizing the need for dietary strategies that enhance performance and recovery. Of 10 studies included, CBT emerged as the most common approach for behavior change. Dietitians were the primary providers of NC interventions and the intervention duration varied widely, ranging from a few weeks to several years. Most studies demonstrated good quality, with minimal bias except for limitations in randomization. NC interventions effectively improved participants' nutritional knowledge and dietary habits, potentially leading to better individual health outcomes, including those with eating disorders. This broadens the scope of NC, displaying its versatility and importance across different sectors of the population.

Despite the clear benefits demonstrated by these studies, there remains a need for more research to fine-tune counseling strategies and maximize their effectiveness. The integration of NC into standard healthcare practices, supported by strong scientific evidence, can significantly enhance the management of metabolic health and lifestyle-related conditions.

The future of NC lies in its ability to adapt to individual needs and leverage technology to improve accessibility and adherence. Continued research and innovation in this field will be essential in addressing the growing epidemic of lifestyle-related diseases.
